# Active Suspension Control for Improved Ride Comfort and Vehicle Performance Using HHO-Based Type-I and Type-II Fuzzy Logic

**DOI:** 10.3390/biomimetics10100673

**Published:** 2025-10-07

**Authors:** Tayfun Abut, Enver Salkim, Harun Tugal

**Affiliations:** 1 Department of Mechanical Engineering, Mus Alparslan University, 49250 Muş, Türkiye; 2 Department of Electronics and Automation, Mus Alparslan University, 49250 Muş, Türkiye; e.salkim@ucl.ac.uk; 3 Department of Electronic and Electrical Engineering, University College London (UCL), Torrington Place, London WC1E 7JE, UK; 4 AI and Robotics Collaborations (ARC), UK Atomic Energy Authority (UKAEA), Culham Campus, Abingdon OX14 3DB, Oxfordshire, UK; harun.tugal@ukaea.uk

**Keywords:** active control, Type I FLC, Type II FLC, Harris Hawks Optimization (HHO) algorithm

## Abstract

This study focuses on improving the control system of vehicle suspension, which is critical for optimizing driving dynamics and enhancing passenger comfort. Traditional passive suspension systems are limited in their ability to effectively mitigate road-induced vibrations, often resulting in compromised ride quality and vehicle handling. To overcome these limitations, this work explores the application of active suspension control strategies aimed at improving both comfort and performance. Type-I and Type-II Fuzzy Logic Control (FLC) methods were designed and implemented to enhance vehicle stability and ride quality. The Harris Hawks Optimization (HHO) algorithm was employed to optimize the membership function parameters of both fuzzy control types. The system was tested under two distinct road disturbance inputs to evaluate performance. The designed control methods were evaluated in simulations where results demonstrated that the proposed active control approaches significantly outperformed the passive suspension system in terms of vibration reduction. Specifically, the Type-II FLC achieved a 54.7% reduction in vehicle body displacement and a 76.8% reduction in acceleration for the first road input, while improvements of 75.2% and 72.8% were recorded, respectively, for the second input. Performance was assessed using percentage-based metrics and Root Mean Square Error (RMSE) criteria. Numerical and graphical analyses of suspension deflection and tire deformation further confirm that the proposed control strategies substantially enhance both ride comfort and vehicle handling.

## 1. Introduction

The purpose of suspension systems in automobiles is to improve comfort by reducing the vibrations that passengers in the vehicle are exposed to while driving and to isolate the effects of vibrations caused by distortions in the road profile. To achieve this, Active Suspension Systems (ASS), which offer effective solutions for suppressing vibrations, are used [1,2,3]. The results obtained in both the simulation and test environment from a hydraulic actuated quarter car suspension system moving under the effects of Coulomb friction were compared by using nonlinear control laws and observer-based nonlinear controllers [4]. Active control of the quarter car system (QCS) using the Fuzzy Logic Control (FLC) method was put forward by Cherry and Jones [5]. The control of the QCS through the Linear Quadratic Gaussian (LQG) method was conducted in a simulation environment [6]. D’Amato and Viassolo simulated the active control of the QCS with a Genetic Algorithm (GA) optimization-based FLC controller with two different road inputs [7]. The active control of the QCS was controlled via the FLC method [8]. Three different road inputs were applied. It was supported by graphical results showing that the method designed and applied in the simulation environment increased driving performance. Al-Holou et al. [9] suggested active control with the FLC method based on sliding mode neural network inference for the QCS and implemented it in the simulation environment. The approach was contrasted with sliding mode control, sliding mode fuzzy logic control, and the passive form of the system. The proposed method was shown to provide high ride comfort and handling qualities.

Abdelhady and Alhasan suggested a neural fuzzy control scheme for active control and put it into practice in a simulation [10]. This approach contrasts with the FLC and Linear Quadratic Regulator (LQR) approaches. In the study, it was revealed that improvements made under a medium-quality road surface and 30 m/s speed, ISO-weighted body acceleration, and dynamic tire deflection provided a reduction of 17% and 20% in RMS values, respectively. Huang and Lin conducted active control of the QCS in a test environment with a self-adjusting adaptive control method with a fuzzy control loop to compensate for the modeling error [11]. The outcomes illustrated that the method decreased the oscillation amplitude of the vehicle body and improved ride comfort. It was shown that the combination of adaptive and fuzzy control strategies only had a significant improvement in suppressing position oscillation and reducing acceleration power spectral density compared to adaptive control and passive systems. The active control of the QCS was conducted by Sharkawy with FLC and Adaptive Fuzzy Control (AFC) methods [12]. Two different road inputs were used in the study. The methods were compared with the LQR control method and the passive version of the system. According to the graphical results, the AFC method provided better results.

Gao et al. [13] presented the control of a hydro-pneumatic ASS based on a nonlinear quarter car model using the Proportional, Integral, and Derivative (PID) control method. Active suspension control was performed by using the Bathfp software package. Zhang et al. [14] presented the simulation of the QCS with a dynamic sliding-mode controller with fuzzy adaptive adjustment for the ASS. According to the simulation results, the suggested controller outperformed the traditional sliding-mode controller’s Linear Quadratic Gaussian control strategy in terms of vibration isolation of the vehicle body. Cao et al. [15] controlled the quarter car system by using an intermittent Type-II FLC. The lower and upper limits of the membership functions were acquired with the AFC from the least mean squares optimal algorithm. The method provided better results compared to the typical FLC and the passive state of the system. Tusset et al. [16] performed the active control of the quarter car system with the FLC method. In the study where the FLC method was suggested and implemented for the control of the QCS, the methods were compared by using PID control management. It reduced body acceleration and increased passenger comfort [17]. Lin et al. [18] performed the control of the quarter car system by using indirect adaptive interval Type-II fuzzy neural network control sliding mode control (Interval Type-II FNNSMC) and Type-I FNNSMC methods. The results obtained by the Interval Type-II FNNSMC method provided better results compared to the Type-I FNNSMC method.

The modeling of an ASS and intermittent Type-II FLC method was performed by Bijan et al. [19]. Type-I and Type-II Fuzzy control methods, the Integral of Absolute Error (IAE) and Integral of Absolute U (IAU), and U control signals were compared to each other numerically. The success of the intermittent type-II fuzzy logic control method was supported with numerical results. Lin and Lia performed active control of the quarter car system by utilizing intelligent control methods [20]. In the simulation, a self-organizing fuzzy controller (SOFC) and hybrid self-organizing fuzzy and radial basis-function neural-network controller (HSFRBNC) methods were applied and compared with each other with graphical and numerical results. The HSFRBNC method produced better results than the SOFC method. Liu et al. [21] suggested a reliable fuzzy H∞ control controller for ASSs with actuator delay and failure and implemented it in the simulation. The method was supported with graphical and numerical results. Hurel et al. [22] obtained fuzzy logic scaling factors with the Particle Swarm Optimization (PSO) for active control of the QCS. Input and output scaling factors of a PSO and PD-type fuzzy controller were acquired. Sprung mass acceleration was defined as a function of the PSO algorithm. The road disturbance attenuation using the optimized fuzzy controller was 81% for a square pulsed road and 51.60% for a random road.

In the study of Kalaivani et al. [23], a hybrid Differential Evolution-based Biogeography-Based Optimization (DEBBO) was suggested for the tuning of an FLC controller implemented on a QCS. It was seen that the suggested controller provided better results than the PID controller, DEBBO-based PID (DEBBOPID), and FLC. Emam used a Fuzzy-PID controller to control the suspension system using a quarter-car model [24]. The methods were compared with each other and their passive status. The study revealed that the value of suspension of working space was reduced by 30.6% with the sprung mass displacement, which improved ride comfort. In addition, sprung mass acceleration decreased by 11.8%, and dynamic tire load was reduced by 18.3%. Divekar and Mahajan modeled the quarter car suspension system and used a self-tuning fuzzy PID control method [25]. The proposed method was contrasted with the PID control and the passive state of the system. The Fuzzy-PID method was found to have less overshoot and settling time compared to the passive PID controller. Abougarair and Mahmoud performed active control of the QCS with PD, LQR, and Fuzzy Logic Tune PD controller methods [26]. The results revealed that the three proposed control techniques were effective in controlling a car but that the Fuzzy Logic Tune PD controller performed better in reducing body displacement, magnitude of suspension travel, and settling time contrasted with the other two controllers. Nagarkar et al. [27] performed active control of the quarter car system with two degrees of freedom with GA-based PID and Fuzzy control methods. With the GA-based multi-objective optimization method, the P, I, and D parameters of the PID controller, the range of the input-output membership functions of the Fuzzy control method, and the scaling parameters were tuned. The results of numerical and graphical simulations showed that the FLC method further improved ride comfort in GA-based FLC and PID control methods.

Lin et al. [28] utilized the QCS in the simulation environment with the proportional differential sliding mode observer-based Fuzzy sliding mode control method. According to the simulation results, the system could increase working stability and driving safety. Hung et al. [29] applied the active control of the QCS in the simulation environment with the FLC method. When the proposed method was compared with the passive state of the system, it was seen that the improvement in ride comfort was approximately 50%. Ahmed actively controlled the QCS by utilizing the Fuzzy PID and LQR control methods [30]. Compared to the Fuzzy-PID control method LQR, it was shown that the sprung mass velocity and the sprung mass acceleration parameter were reduced, and less overshoot and a very small settling time were achieved. Matrood and Nassar used the modified PID control method in the active control of a QCS [31]. The method was contrasted with the traditional PID control. The dynamic responses of the system, such as acceleration, speed, and suspension movement, were presented with numerical and characteristic results, which were improved when the modified PID controller was utilized.

In a study, the ASS was controlled in a simulation environment with the fuzzy model predictive controller method [32]. The suggested method was contrasted via the passive and semi-active status of the system by using bump and sinusoidal road inputs. The results obtained by utilizing PID, PID-LQR, and Fuzzy-PID control methods in the control of quarter and semi-active suspension systems were displayed by Munawwarah and Yakub [33]. When the methods were compared, it was seen that Fuzzy-PID reduced vibration in terms of vehicle body displacement, vehicle body acceleration, and wheel deviation in the rear and front suspension systems, but unsatisfactory driving and poor road holding in the rear suspension system were obtained when contrasted with PID-LQR and PID control methods. An adaptive harmonic control for a quarter car’s active suspension was suggested by Nichiţelea and Unguritu, who then implemented it in a simulation environment [34]. In the study, in which PI controller, H∞ controller, and model predictive control methods were also used, when the simulation results were compared, it was revealed with numerical and graphical results that the proposed method (adaptive harmonic control method) produced better results in certain performance criteria. Wang et al. [35] implemented the Sliding Mode Control (SMC) parameters to the quarter car suspension system with Type-II and Type-I Fuzzy. The Type II SMC method provided better results in the system where two different road inputs were applied.

Mahmoodabadi and Javanbakht performed active control of the quarter car model via the optimum AFC controller by using the Gravitational Search Algorithm (GSA) [36]. The proposed method was compared with three models. Successful numerical results were obtained compared to the traditional controllers. Robert et al. [37] performed the control of a suspension system based on the quarter car model with an FLC controller. The simulation results showed that it performed better than the traditional controller in terms of body displacement and body acceleration. Han et al. [38] applied and analyzed the adaptive fuzzy PID control method on the QCS in a simulation. When the suggested method was contrasted via the PID control method and the passive state of the system, it was seen that the performance of the vehicle increased. Based on the fuzzy model of Takagi–Sugeno (T–S), a new optimization control algorithm for active suspension systems of vehicles was suggested by Zhang and Dong [39]. Online optimization of the membership functions (MFs) of the suspension systems was implemented in the QCS. Comparative results were presented. The active control of the QCS was performed by Abut and Salkim by using LQR, PSO-based Fuzzy Logic, and Fuzzy Linear Quadratic Regulator control methods [40]. The methods were contrasted based on the passive system and the studies in the literature. According to the comparisons conducted by using the integral time absolute error performance error criterion, it was revealed that the performance of the suspension system rose compared to the passive system by 84.2% in terms of vehicle body movement, 90% vehicle acceleration, 84.5% suspension deflection, and 86.7% tire deflection. In the study, the increase in road handling and ride comfort compared to the current literature was also shown with numerical data.

Nguyen controlled the quarter car suspension system in the simulation via the sliding mode-fuzzy PID control method [41]. In the study, it was observed that the peak values of vehicle body acceleration and displacement were significantly decreased compared to other methods, and ride comfort was increased. Nguyen simulated the active control of the quarter car suspension system with the Adaptive Fuzzy-Sliding Mode Proportional-Integral (AFSP) method [42]. The AFSP method consists of a linear controller, PI (Proportional-Integral), a nonlinear controller, and SMC signals. This method was compared with SMC and passive status. It was determined that the acceleration and displacement values did not exceed 80% and 20%, respectively, compared to the AFSP method used in the study, compared to the passive suspension system. Maximum values and mean values of sprung mass displacement and acceleration determined according to Root Mean Square (RMS) criteria were greatly reduced compared to other methods. Zhao and Gu controlled the quarter car system by using a hybrid optimization detection that obtained the weight coefficients of the LQR control method [43].

The hybrid algorithm is an algorithm that unites the advantages of the PSO algorithm and the GA. In the study, the method was contrasted with the passive status of the system and traditional LQR control method, and it was seen that the RMS of the sprung mass acceleration, deflection, and tire dynamic load under LQR control with optimization decreased by 22.7%, 53%, and 68% in Case 1, and 74%, 71%, and 90% in Case 2, compared with the LQR control without optimization. Wu and Su proposed a BP neural network controller based on Q-learning algorithm optimization (QBP-PID) for active control of the QCS [44]. It was revealed that the active suspension using the QBP-PID control algorithm significantly improved the driving stability and comfort of the vehicle compared to the conventional passive suspension, PID control suspension, and BP-PID control suspension. Kharola et al. [45] presented the results obtained using PID and adaptive neuro-fuzzy inference system (ANFIS)-based control methods for active control of the QCS. In the study comparing the results of both controllers, it was revealed that the ANFIS controller illustrated superior performance in terms of settling time and overshoot responses.

Mustafa and Wang proposed a new adaptive fuzzy logic control method for active control of nonlinear quarter car suspension systems based on time delay [46]. The suggested method was contrasted with time delay control, PID, and a conventional passive system for the suspension system under different road inputs, and it was observed that the suggested method showed superior performance. Abut et al. [47] designed LQG and Fuzzy Linear Quadratic Gaussian control methods and utilized them for active control to improve vehicle handling and passenger comfort and improved car body motion, vehicle acceleration, suspension deflection, and tire deflection by about 88.2%, 91.5%, 88% and 89.4%, respectively. Lopes et al. [48] obtained PID control parameters using the giant armadillo optimization algorithm and implemented them in a quarter suspension system. It has been shown by numerical simulations that the proposed method reduces 36.36% and 39.34% in the mean absolute error and root mean squared error, respectively, compared to the Ziegler and Nichols method. Zhang et al. [49] proposed and implemented a neuroadaptive control method based on a biologically inspired reference model and a fully actuated system approach for indefinite active suspension systems with input dead zones. The results obtained illustrate that the designed control method achieves significant improvements in transient performance, with a 31% or more improvement, and saves 32% or more energy control compared to existing control methods. A summary of the controllers and optimizers found in the literature review provided in the article is given in Table 1.

This article discusses the adoption of active control methods. The suspension model was formed with the Lagrange–Euler method, and Type-I and Type-II Fuzzy Logic control methods were designed and applied to increase vehicle road handling and comfort. Two different road inputs were applied to the system. Optimum coefficients of Type-I and Type-II Fuzzy membership functions were optimized using the HHO algorithm. The methods were contrasted with each other based on Percentage and the RMSE performance criteria. Suspension deflection and tire deformations are given in the graphs. This study explores active control methods designed to reduce vibrations in car suspension systems. It has been observed that conventional passive suspension systems are not effective enough in minimizing vibrations, which in turn fails to enhance ride comfort. To address this limitation, the HHO algorithm is applied to optimize the membership functions of Type-I and Type-II Fuzzy Logic control. These optimal coefficients are then implemented in the ASS, and to enable the system with the ability to decide itself. Consequently, notable enhancements in performance were attained in contrast to the passive suspension system.

The contributions of this study are briefly presented below.

- The first main contribution of our study is to optimize the membership functions of the Type-II Fuzzy Logic Control method using the Harris Hawks Optimization (HHO) algorithm and apply these optimal coefficients to the active suspension system, enabling the system to autonomously make control decisions.
- The second contribution is the novel use of HHO-based Type-I and Type-II Fuzzy Logic control methods together, followed by a comprehensive comparison between the passive system state and the controlled results.
- The third contribution is the definition of performance indices for the applied methods and their comparison with each other and with existing studies in the literature.

Although many studies have explored fuzzy control systems and metaheuristic optimization separately, there remains a research gap regarding the integrated use of the HHO algorithm for tuning Type-II fuzzy controllers, specifically in active suspension systems. This study addresses this gap by proposing a novel hybrid approach that combines bio-inspired optimization with advanced fuzzy control techniques. Therefore, we believe this work offers an original contribution to the field and advances the development of intelligent control strategies for automotive suspension systems. This paper’s remaining content is Section 2 presents the System Modeling of Vehicle Suspension. Section 3 describes the design of the HHO-based Type-I and Type-II Fuzzy Logic control methods. Section 4 reviews Results and Discussion of the Simulation. The study’s numerical and graphical findings are presented in this section. Additionally, a table of comparisons between the literature and its interpretation is presented after this chapter. The conclusion is finally summarized in Section 5. The results of the article are examined and interpreted in this part. Furthermore, recommendations for using the approach and details of upcoming research on the approach are provided at the conclusion of this section.

This study presents a novel control strategy for active suspension systems using the nature-inspired the HHO algorithm. The HHO is a bio-inspired metaheuristic that mimics the cooperative and strategic hunting behavior of Harris hawks. By mathematically modeling these behaviors, the algorithm effectively optimizes control parameters to achieve a balance between ride comfort and road handling. Simulation results demonstrate that the HHO-based controller outperforms conventional methods, offering enhanced vibration reduction and improved road contact. The findings highlight the potential of biomimetic algorithms in addressing complex engineering problems, particularly in the development of advanced automotive technologies.

## 2. System Modeling of Vehicle Suspension

**Figure 1:**
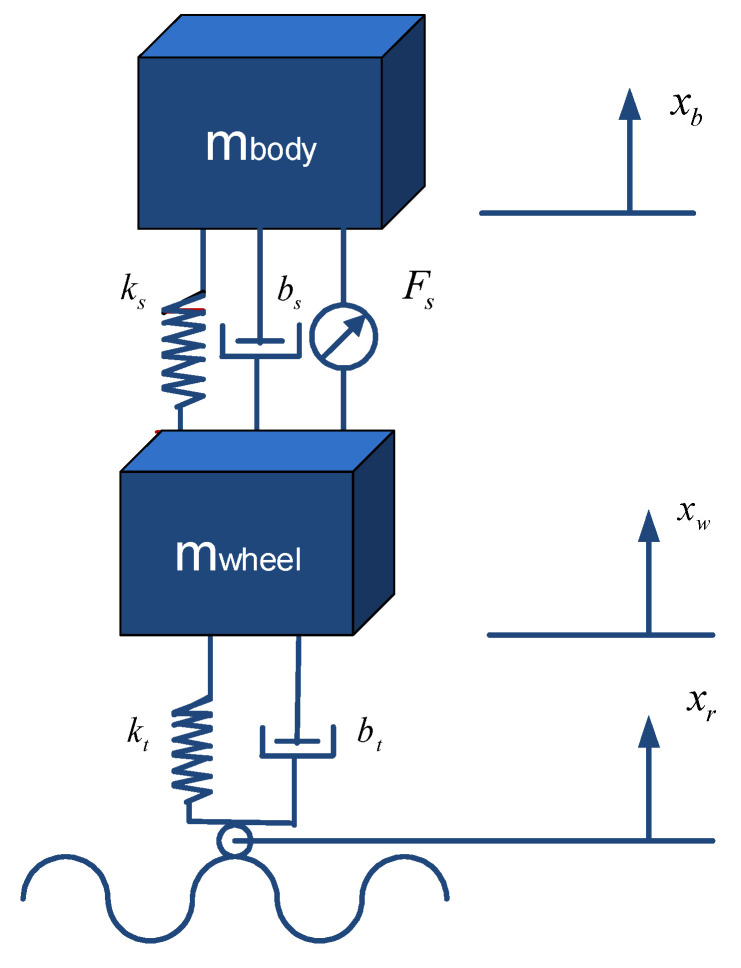
The quarter-car system.

The quarter car system with two degrees of freedom was acquired by utilizing the Lagrange–Euler method and is presented in Figure 1. The model had vertical vibration movement and was symmetrically divided into four pieces; however, the wheel and chassis pitch and roll movements were not taken into account. Below are the model equations:

m_body_ (*m_b_*) represents the mass of a quarter of the vehicle body, m_wheel_ *(m_w_*) represents the mass of the wheel assembly, *k_s_* represents the sprung coefficient of the suspension system, *b*_s_ represents the damper coefficient of the suspension system, *k_t_* represents the sprung coefficient of the tire, and *b_t_* represents the damper coefficient of the tire. In addition, the state variables illustrate the displacement (*x_w_*) movements of the body (*x_b_*) and the wheel assembly, while the input variables illustrate the *x_r_* road distortion and the control force exerted by the active element implemented between the *F_s_* body and the wheel.

(1) $$m_{b}{\ddot{x}}_{b}+b_{s}\left({\dot{x}}_{b}-{\dot{x}}_{w}\right)+k_{s}\left(x_{b}-x_{w}\right)-F_{s}=0$$

(2) $$m_{w}{\ddot{x}}_{w}+b_{t}\left({\dot{x}}_{w}-{\dot{x}}_{r}\right)+k_{t}\left(x_{w}-x_{r}\right)-b_{s}\left({\dot{x}}_{b}-{\dot{x}}_{w}\right)-k_{s}\left(x_{b}-x_{w}\right)-F_{s}=0$$

(3) $${\ddot{x}}_{b}=\frac{-1}{m_{b}}\left(b_{s}\left({\dot{x}}_{b}-{\dot{x}}_{w}\right)+k_{s}\left(x_{b}-x_{w}\right)-F_{s}\right)$$

(4) $${\ddot{x}}_{w}=\frac{-1}{m_{w}}\left(b_{t}\left({\dot{x}}_{w}-{\dot{x}}_{r}\right)+k_{t}\left(x_{w}-x_{r}\right)-b_{s}\left({\dot{x}}_{b}-{\dot{x}}_{w}\right)-k_{s}\left(x_{b}-x_{w}\right)-F_{s}\right)$$

(5) $$x_{1}=x_{b};x_{2}=x_{w};x_{3}={\dot{x}}_{b};x_{4}={\dot{x}}_{w};$$

$${\dot{x}}_{1}=x_{2}$$

$${\dot{x}}_{2}=\frac{-1}{m_{b}}\left(b_{s}\left(x_{3}-x_{4}\right)+k_{s}\left(x_{1}-x_{2}\right)-F_{s}\right)$$

$${\dot{x}}_{3}=x_{4}$$

(6) $${\dot{x}}_{4}=\frac{-1}{m_{w}}\left(b_{t}\left(x_{4}-{\dot{x}}_{r}\right)+k_{t}\left(x_{2}-x_{r}\right)-b_{s}\left(x_{3}-x_{4}\right)-k_{s}\left(x_{1}-x_{2}\right)-F_{s}\right)$$

This is the state-space version ($\dot{x}=Ax+Bu$.) of the dynamic model of the linear system:

(7) $$\left[\begin{array}{c} {\dot{x}}_{1}\\ {\dot{x}}_{2}\\ {\dot{x}}_{3}\\ {\dot{x}}_{4} \end{array}\right]=\left[\begin{array}{ccc} 0 & 1 & \begin{array}{cc} 0 & 0 \end{array}\\ \frac{-k_{s}}{m_{b}} & \frac{-b_{s}}{m_{b}} & \begin{array}{cc} \frac{k_{s}}{m_{b}} & \frac{b_{s}}{m_{b}} \end{array}\\ \begin{array}{c} 0\\ \frac{k_{s}}{m_{w}} \end{array} & \begin{array}{c} 0\\ \frac{b_{s}}{m_{w}} \end{array} & \begin{array}{cc} \begin{array}{c} 0\\ \frac{-k_{s}-k_{t}}{m_{w}} \end{array} & -\begin{array}{c} 1\\ \frac{b_{s}}{m_{w}} \end{array} \end{array} \end{array}\right]\left[\begin{array}{c} x_{1}\\ x_{2}\\ x_{3}\\ x_{4} \end{array}\right]+\left[\begin{array}{cc} 0 & 0\\ 0 & \frac{1}{m_{b}}\\ \begin{array}{c} 0\\ \frac{k_{t}}{m_{w}} \end{array} & -\begin{array}{c} 0\\ \frac{1}{m_{w}} \end{array} \end{array}\right]$$

(8) $$y=\left[\begin{array}{ccc} 1 & 0 & \begin{array}{cc} 0 & 0 \end{array}\\ 1 & 0 & \begin{array}{cc} -1 & 0 \end{array}\\ \frac{-k_{s}}{m_{b}} & \frac{-b_{s}}{m_{b}} & \begin{array}{cc} \frac{k_{s}}{m_{b}} & \frac{b_{s}}{m_{b}} \end{array} \end{array}\right]\left[\begin{array}{c} x_{1}\\ x_{2}\\ x_{3}\\ x_{4} \end{array}\right]+\left[\begin{array}{cc} 0 & 0\\ 0 & 0\\ 0 & \frac{1}{m_{b}} \end{array}\right]$$

The variable *y* represents the output of the system, which varies depending on the inputs and internal states of the system. The transfer function given in (9) illustrates the transfer function to the dynamics of the actuator utilized in the ASS. For the modeling and simulation of the passive suspension system, constant spring and damper parameters and linear differential equations are used in the quarter-vehicle suspension model. The same model parameters are also employed for the ASS. Equations (10) and (11) were used to obtain road entry profiles (Road 1 and Road 2), respectively. In this study, where the measurement of body acceleration ${\ddot{x}}_{b}$ was utilized as feedback, the control of the quarter car ASS was given.

(9) $$H_{act}\left(s\right)=\frac{1}{1/60\;s+1}$$

(10) $${road}_{1}\left(t\right)=0.05\times \left[1-cos\left(8\pi t\right)\right]$$

(11) $${road}_{2}\left(t\right)=0.03\times \left[1-cos\left(2\pi t\right)\right]$$

## 3. Design and Implementation of System Controllers

**Figure 2:**
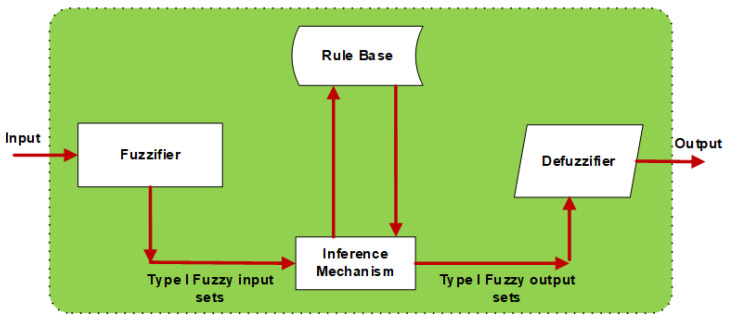
Structure of Type-I fuzzy logic system.

Controllers were created to improve the vehicle’s performance and passenger comfort. Low vibration and a robust system are the main objectives of the controllers designed for active suspension systems. Type-I and Type-II FLC methods were utilized in the quarter car system. The design processes of these proposed methods will be discussed in detail. The fuzzy logic algorithm is a method proposed by Zadeh. It consists of five phases [52,53]. The general structure of the Type-I Fuzzy logic algorithm is given in Figure 2. The first phase is fuzzification, where input variables are converted into a fuzzy set. In the second phase, rule tables, membership functions, and rule bases are created.

**Figure 3:**
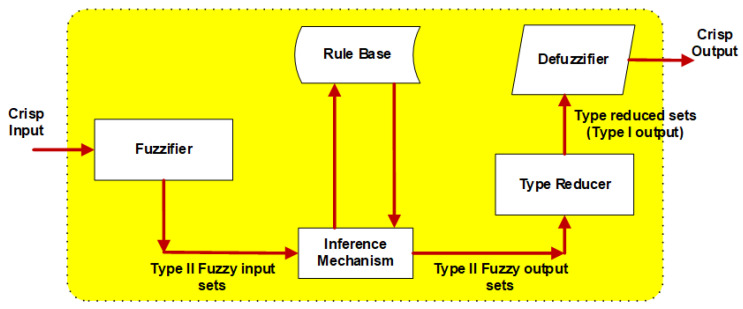
Structure of Type-II fuzzy logic system.

This rule base is composed of IF-THEN rules obtained from the verbal expressions of experts who know the system. The third phase is the inference mechanism. In the fourth phase, membership functions and fuzzy clusters are described. The final phase is defuzzification, where a fuzzy set is transformed into a net value for output. Type-I Fuzzy control is a classical fuzzy logic-based control method used in systems containing uncertainty and variability. This method provides control of the system by using membership functions to define the relationships between input variables and control outputs. Type-II fuzzy logic is the approach emerging to minimize the effects of uncertainties in Type-I fuzzy logic systems [54,55]. Uncertainties can be modeled with Type-II fuzzy logic since their membership functions are fuzzy. The membership functions in this method are three-dimensional. All parameters are shown in the third dimension of Type-II fuzzy logic clusters, such as uncertain words obtained in fuzzy rules, information obtained from disagreeing experts, noisy measurements running the system, and noisy data setting the parameters [56,57,58]. Type-II Fuzzy control is a method that allows uncertainties to be handled in a more complex structure. In this method, there is another layer of uncertainty over membership functions, which offers more flexibility and precision. Type-II Fuzzy enables the system to operate more accurately and reliably under various conditions. The structure of the Type-II Fuzzy logic system is illustrated in Figure 3.

As seen in Figure 3, the sharp input is converted into a fuzzy cluster by the fuzzifier. It is similar to the rule base and inference mechanism structure. Another difference from Type-I is that there is a Type reducer. Accordingly, the structure of the rules is used in the same way as Type-I, with the only difference being that the membership functions in Type-II are a range of Type-II. The use of fuzzy logic control methods in ASS studies continues in the literature today [50,51,59]. The coefficients of the membership functions used in the proposed methods were obtained by using the HHO method [60]. The HHO method is a swarm-based optimization method inspired by nature [61,62]. The main logic of the method is that it was designed based on the cooperative behaviors and chasing styles of Harris hawks. The three primary components of the HHO algorithm are exploration, the change from exploration to exploitation, and actual exploitation. The initial stage of imitating Harris hawk behavior during prey search is called the exploration phase (Equation (12)).

(12) $$\begin{array}{c} x\left(t+1\right)=\\ \left\{ \;\begin{array}{c} x_{rand}\left(t\right)-r_{1}\left|x_{rand}\left(t\right)-2r_{2}x\left(t\right)\right|\;\;\;\;\;\;\;\;\;\;\;\;\;\;\;\;\;\;\;\;\;\;\;\;\;\;\;\;\;q\geq 0.5\\ \left(x_{rabbit}\left(t\right)-x_{m}\left(t\right)\right)-r_{3}\left(LB+r_{4}\left(UB-LB\right)\right)\;\;\;\;\;\;q<0.5 \end{array}\;\;\;\;\;\;\;\;\;\;\;\;\;\;\;\;\;\;\;\;\;\;\;\;\;\;\;\;\;\;\;\;\;\;\;\;\;\right. \end{array}$$

where $x\left(t\right)$ and $x\left(t+1\right)$ denote the hawk position at the current iteration and the next iteration, respectively; $r_{1}$, $r_{2}$, $r_{3}$, $r_{4}$, and q are varying random numbers in the range [0, 1]; $x_{rabbit}\left(t\right)$ and $x_{rand}\left(t\right)$ denote the rabbit position (best position) and the randomly selected hawk position, respectively. Additionally, *LB* and *UB* denote lower bands and upper bands, respectively, and $x_{m}\left(t\right)$ is the average position of the Harris Hawk and can be calculated from Equation (13) below.

(13) $$x_{m}\left(t\right)=\frac{1}{N}\sum_{i=1}^{N}x_{i}\left(t\right)$$

where $x_{i}\left(t\right)$ is the position of each hawk at iteration *t* and *N* is the number of all hawks. The second phase is the transition phase to exploitation. The energy of falcons decreases during chasing and hunting. The energy of prey can be defined as follows:

(14) $$E=2E_{0}\left(1-\frac{t}{T}\right)$$

where $E_{0}$ is the initial energy, *T* is the total number of iterations and *E* is the escaped energy. If $\left|E_{0}\right|\geq 1$, the hawks are in the exploration phase, and if $\left|E_{0}\right|<1$, the hawks are in the exploitation phase, that is, the hawks are chasing and attacking a rabbit. The last phase is the exploitation phase, which mainly aims to improve local solutions from the solutions found earlier. The last phase considers both the possibility of escape and the energy level of the prey. Prey always tries to escape from its predators. Depending on the prey’s escape behavior, i.e., escape chance *r* and escape energy *E*, hawks have four possible chase-attack strategies. If the escape chance r≥0.5, the prey is assumed to escape successfully, and if r<0.5, the escape is assumed to fail. Whatever the prey does, it is surrounded by hawks from different directions, softly or hard, depending on its remaining energy. Hence, the hawks perform soft encirclement when $\left|E_{0}\right|\geq 0.5$ and hard encirclement when $\left|E_{0}\right|\geq 0.5$. These strategies are described in the following subsections. For the case r≥0.5 and $\left|E_{0}\right|\geq 0.5$, the hawks will follow a soft flanking strategy formulated in Equations (15) and (16).

(15) $$x\left(t+1\right)=∆x\left(t\right)-E\left|{Jx}_{rabbit}\left(t\right)-x\left(t\right)\right|$$

(16) $$∆x\left(t\right)=x_{rabbit}\left(t\right)-x\left(t\right)$$

where $∆x\left(t\right)$ is the difference between the current position of the rabbit and the hawks at iteration *t*, $J=2\left(1-r_{5}\right)$ is the jumping power of the rabbit when escaping, and $r_{5}$ is an arbitrary number in the range (0, 1). When r≥0.5 and $\left|E\right|<0.5$, the hawks will follow the hard encirclement strategy formulated in Equation (16).

(17) $$x\left(t+1\right)=x_{rabbit}\left(t\right)-E\left|∆x\left(t\right)\right|$$

When r<0.5 and $\left|E_{0}\right|\geq 0.5$, the hawks will follow the soft encirclement strategy with fast dives formulated in Equation (18). Here, *Y* represents the next move decided by the smart hawks under soft encirclement. Hawk dives in this phase are based on the Levy flight (*LF*) pattern formulated in Equation (19).

(18) $$Y=x_{rabbit}\left(t\right)-E\left|{Jx}_{rabbit}\left(t\right)-x\left(t\right)\right|$$

(19) $$Z=Y+S\times LF\left(D\right)$$

where *D* is the dimension and *S* is a random vector. The Levy flight, or LF, is determined by the following formula (Equation (20)):

(20) $$LF\left(x\right)=\frac{u\times \sigma }{{\left|v\right|}^{\frac{1}{\beta }}},\;\;\;\;\sigma ={\left(\frac{\Gamma \left(1+\beta \right)\times sin(\frac{\pi \beta }{2})}{\Gamma \left(\frac{\left(1+\beta \right)}{2}\right)\times \beta \times 2^{\left(\frac{\beta -1}{2}\right)}}\right)}^{\frac{1}{\beta }}$$

Here, *u* and *v* are random values in the range (0, 1) and *β* is a constant value of 1.5. Therefore, the final strategy to update the positions of the hawks at this stage is implemented as follows (Equation (21)):

(21) $$x\left(t+1\right)=\left\{\begin{array}{c} YF\left(Y\right)<F\left(x\left(t\right)\right)\\ ZF\left(Z\right)<F\left(X\left(t\right)\right) \end{array}\right.$$

At each step, only the best *Y* or *Z* is selected as the next location. When r<0.5 and $\left|E\right|<0.5$, the hawks will follow the hard encirclement strategy with fast dives formulated in Equation (22).

(22) $$x\left(t+1\right)=\left\{\begin{array}{c} Y^{\prime }F\left(Y^{\prime }\right)<F\left(x\left(t\right)\right)\\ Z^{\prime }F\left(Z^{\prime }\right)<F\left(X\left(t\right)\right) \end{array}\right.$$

where *Y’* and *Z’* are acquired by the new rules formulated in Equations (23) and (24):

(23) $$Y^{\prime }=x_{rabbit}\left(t\right)-E\left|{Jx}_{rabbit}\left(t\right)-x\left(t\right)\right|$$

(24) $$Z^{\prime }=Y+S\times LF\left(D\right)$$

The method has been detailed in [56,57,58].

Optimizing Type-I and Type-II Fuzzy Logic Membership Functions with the HHO Algorithm steps:

Step 1. Initialization and Parameter Settings:

Initialization parameters are set to initialize the HHO algorithm. Iteration count: The number of generations the algorithm will run is determined.

Population size: The size of the initial population is determined. Exploration and exploitation parameters: Parameters that balance the exploration and exploitation phases in the HHO algorithm.

Step 2. Creating the Initial Population:

In the HHO algorithm, the initial population consists of solution vectors representing the parameters of the membership functions (e.g., alpha, beta, gamma, etc.). This population is usually generated randomly, but optimization is started upon initial conditions.

Step 3. Defining the Fuzzy Logic System:

The configuration of the Type-I and Type-II fuzzy logic systems is performed. At this stage, input and output variables are determined. Membership functions are selected, and their parameters are defined. Triangular, trapezoidal, etc., membership functions can be selected for Type I, and more complex membership functions can be selected for Type II.

Step 4. Optimization with the HHO Algorithm:

The HHO algorithm evaluates the performance of the FLC system for each individual (solution). The performance evaluation is usually done to minimize the output error of the system. Objective function (ITAE): Usually, optimization is achieved by minimizing the error function (e.g., squared error or integral squared error). The Harris Hawks Optimization algorithm updates the position of each individual in the population and uses exploration and exploitation strategies to find the optimal solution.

Step 5. Selection of the Best Solution:

At the end of each generation, the best solution (optimal membership function parameters) in the population is selected. The optimal solution forms the best set of parameters that improve the performance of the fuzzy logic system.

Step 6. Testing and Evaluation with Fuzzy Logic Control System:

Control tests are performed on the active suspension system with the obtained optimum membership function parameters. The output numbers and the system’s sensitivity are used to assess the system’s performance. By contrasting the test findings with those of the passive suspension system, the efficacy of the active control approach is evaluated.

Step 7. Conclusion and Improvement:

At the end of the algorithm, the dynamic performance of the suspension system is observed by fuzzy logic control of the optimal solution. If necessary, further improvements can be made to the HHO algorithm or different optimization techniques can be used.

**Figure 4:**
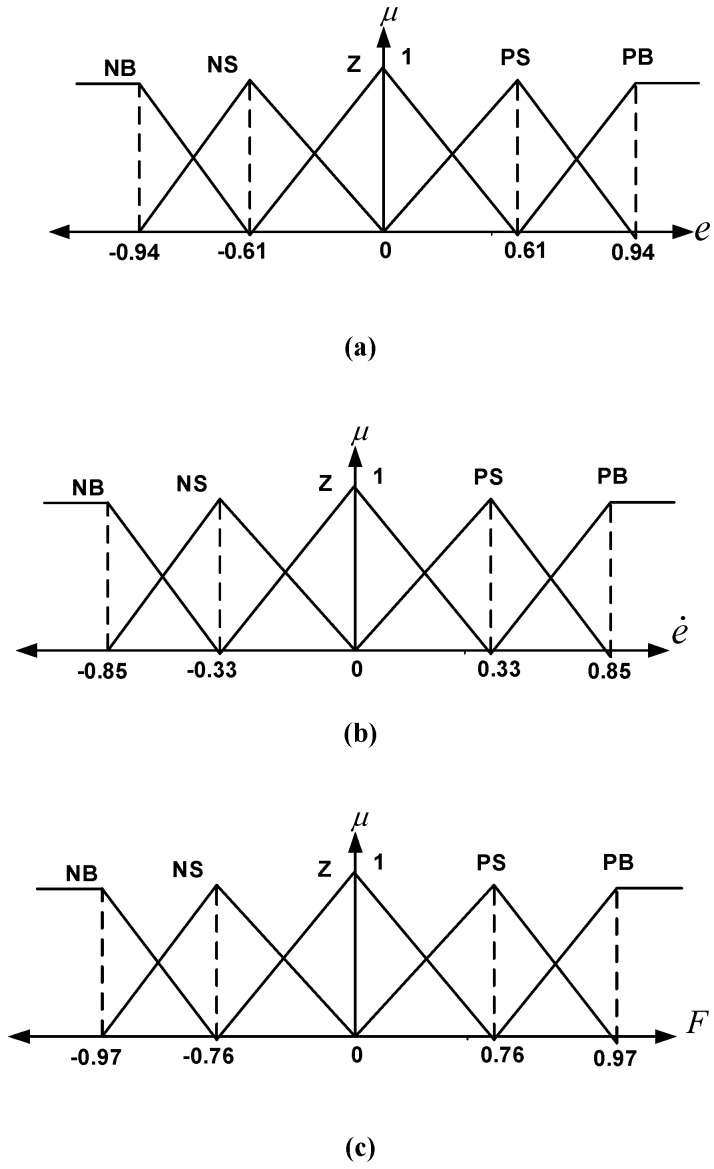
HHO-based Type-I Fuzzy membership functions defined for (**a**) e (**b**) $\dot{e}$ and (**c**) F which are input and output values.

For the HHO algorithm, the *Integral of the Time-Weighted Absolute Error (ITAE)* was utilized as the objective function to minimize the errors acquired as a result of the operation of the system.

(25) $$Object\;function=ITAE=\int_{0}^{t}t\left|e\right|dt$$

e shows the position error of the car. In the optimization routine, during the testing phase, the performance of each proposed solution (e.g., fuzzy controller parameters) is evaluated through simulations. The performance is measured based on the defined fitness function (ITAE). This fitness value is then used by the optimization algorithm’s decision-making process to guide the search for better solutions. The process continues until certain stopping criteria are met (e.g., maximum number of iterations or error tolerance). The working time is shown with t. (e) refers to controller error, ($\dot{e}$) refers to the change rate of errors, and F is used for the output value. Triangle-type membership functions were used in the Type-I and Type-II Fuzzy control methods. Triangle-type membership functions and the Mamdani inference method were used for all input and output values in the proposed methods. In the HHO algorithm used in both control methods, optimum values were obtained when the population size was determined as 50 and the maximum iterations as 30. In this study, the LB and UB boundary conditions for both control methods were set to −1 and 1, respectively. In our study, the dynamic equations of the quarter-car suspension system were numerically solved using the 4th-order Runge–Kutta method in the simulations. A time step of 0.001 s was chosen. This method allows for accurate and stable capturing of the model’s dynamic behavior. The HHO algorithm was executed 30 times for each test scenario. The results indicate that the algorithm demonstrates consistent and reliable performance across repeated runs. The triangle-type membership functions and coefficients (respectively, e, $\dot{e,}$ and F) obtained by using the HHO algorithm for the Type-I Fuzzy control method are illustrated in Figure 4a–c. Table 2 presents the rule table formed for the HHO-based Type-I Fuzzy control method.

**Figure 5:**
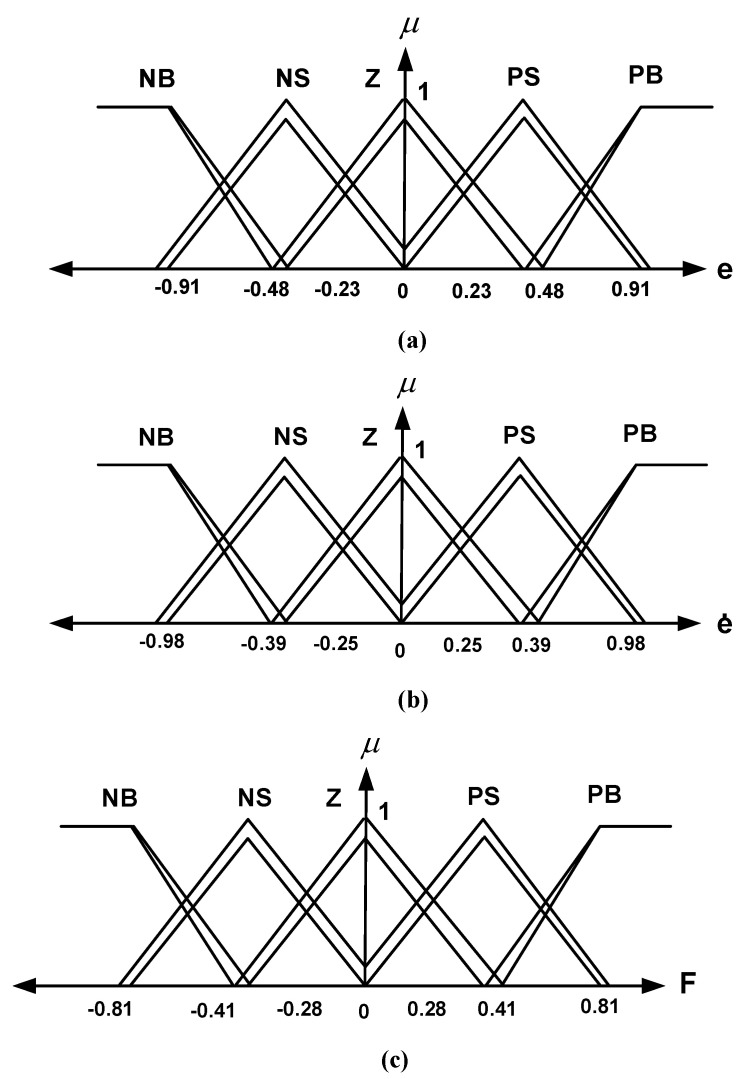
HHO-based Type-II Fuzzy membership functions are defined for (**a**) e, (**b**) $\dot{e}$, and (**c**) F, which are input and output values.

The control variables given in Table 2 and Figure 4 are expressed as $e,\dot{e},F$ error, error change, and force, respectively. NB, NS, Z, PS, and PB refer to Negative Big, Negative Small, Zero, Positive Small, and Positive Big expressions, respectively. The triangle-type membership functions and coefficients (e, $\dot{e,}$ and F, respectively) obtained by using the HHO algorithm for the Type-II Fuzzy control method are shown in Figure 5a–c. Table 3 shows the rule table formed for the HHO-based Type-II Fuzzy control method.

The control variables given in Table 3 and Figure 5 are expressed as e, $\dot{e}$, F error, error change, and force, respectively. NB, NS, Z, PS, and PB expressions refer to Negative Big, Negative Small, Zero, Positive Small, and Positive Big expressions, respectively.

## 4. Results and Discussion

**Figure 6:**
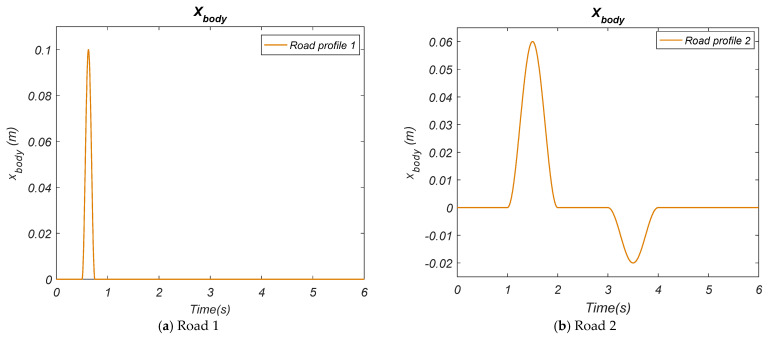
The Road inputs (**a**,**b**).

In this section, the control methods designed for a quarter-car suspension system, and the simulation results of these methods are displayed. The Type-I and Type-II Fuzzy control methods based on the HHO algorithm were designed and implemented to provide active control of the system. The results obtained were analyzed in detail in line with tables and graphs. The primary objective of controlling a car suspension system is to minimize the negative disruptive effects of road input on passenger comfort. The physical parameters of the quarter car suspension system are as follows: vehicle body mass m_b_ = 300 kg, wheel mass m_w_ = 60 kg, suspension sprung hardness k_s_ = 16,000 N/m, tire hardness k_t_ = 190,000 N/m, suspension damper coefficient b_s_ = 1000 Ns/m and suspension damper coefficient b_t_ = 1000 Ns/m. The initial position of the system was taken as x = 0 m, while the simulation time was taken as 6 s. Simulations were performed on a computer with an Intel Core i5-10400 processor, 8 GB of RAM, and Windows 10 Pro operating system. All analyses and simulations were performed using MATLAB/Simulink R2016a. The road inputs are bumps and are shown in Figure 6a,b.

**Figure 7:**
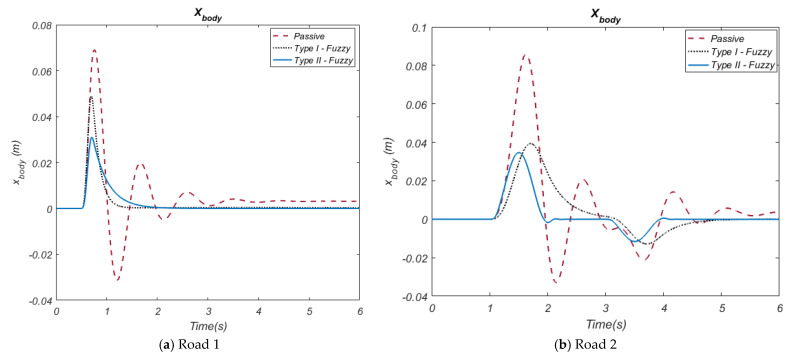
The car body travel.

**Figure 8:**
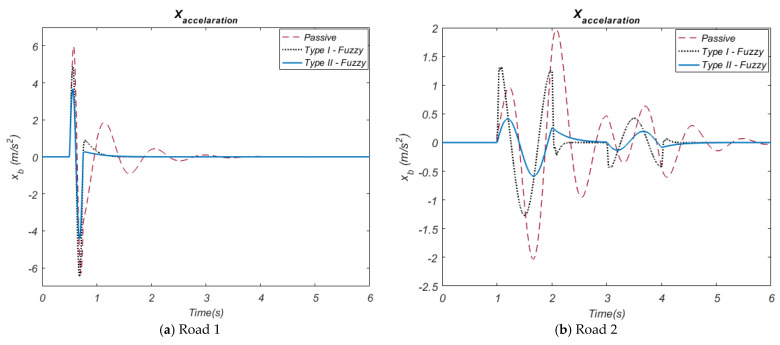
The car body acceleration.

The simulation results obtained by using both the passive state of the suspension system and the active control methods were shown comparatively with the graphs. In this context, the performance of HHO algorithm-based Type-I and Type-II fuzzy control methods were evaluated. Movement of the vehicle body (displacement) and acceleration of the vehicle body are important parameters that directly affect passenger comfort. In terms of both road inputs (Road 1 and Road 2), the simulation results of these parameters are presented in Figure 7 and Figure 8 in comparison with the passive system.

When the vertical displacement motion graph of the vehicle body obtained related to the Road 1 input (Figure 7a) is observed, it is seen that the vertical displacement motion amplitudes of the HHO-based Type-II Fuzzy control method are lower compared to the HHO-based Type-I Fuzzy control method. While the HHO-based Type-II Fuzzy control method provides a vertical displacement movement amplitude of approximately 0.03~0 m, the Type-I Fuzzy control method has a vertical displacement movement amplitude of approximately 0.048~0 m. When the vertical displacement motion graph of the vehicle body obtained related to the Road 2 input (Figure 7b) is examined, it is observed that the vertical displacement motion amplitudes of the HHO-based Type-II Fuzzy control method are lower than the HHO-based Type-I Fuzzy control method. While the Type-II Fuzzy control method provides the vertical displacement movement amplitude of a vehicle body in the range of approximately 0.037~−0.015 m, the Type-I Fuzzy control method has the vertical displacement movement amplitude of a vehicle body in the range of approximately 0.04~−0.018 m. These results reveal that HHO-based Type-II Fuzzy control controls the vertical displacement movement of the vehicle body better.

**Figure 9:**
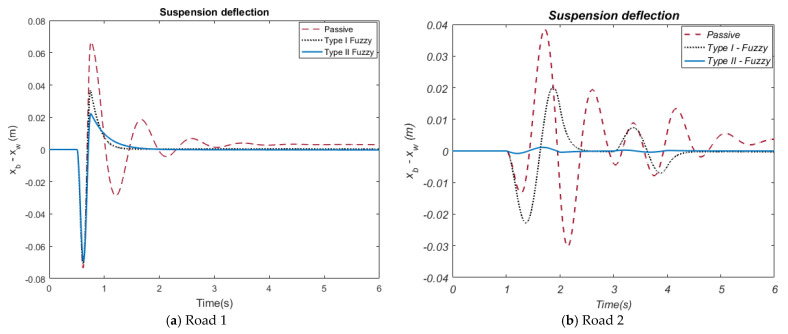
The car suspension deflection.

**Figure 10:**
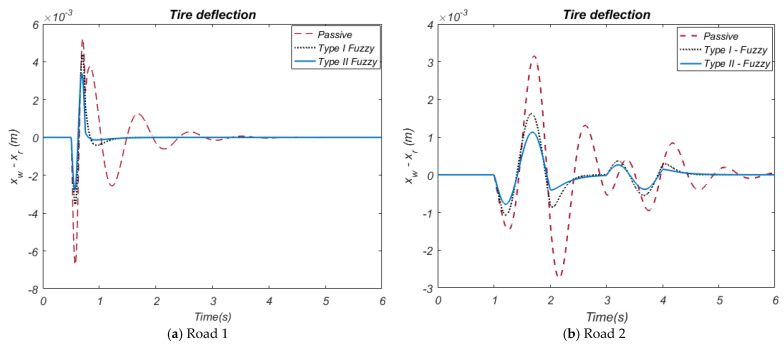
The car tire deflection.

When the vertical displacement motion graphs of the vehicle body given above are examined, it is seen that all these controls methods significantly decrease the vibration amplitude and settling time compared to the passive suspension system. When the acceleration graph obtained related to the Road 1 input (Figure 8a) is examined, it is seen that the acceleration amplitudes of the HHO-based Type-II Fuzzy control method are lower than the HHO-based Type-I Fuzzy control method. The HHO-based Type-II Fuzzy control method provides an acceleration amplitude of approximately 0.38~−0.42 m/s^2^, while the Type-I Fuzzy control method has an acceleration amplitude of approximately 0.50~−0.62 m/s^2^. When the acceleration graph obtained related to the Road 2 input (Figure 8b) is examined, it is observed that the acceleration amplitudes of the HHO-based Type-II Fuzzy control method are lower compared to the HHO-based Type-I Fuzzy control method. While the Type-II Fuzzy control method provides an acceleration amplitude in the range of approximately 0.43~−0.52 m, the Type-I Fuzzy control method has an acceleration amplitude of approximately ±1.25 m/s^2^. These results reveal that HHO-based Type-II Fuzzy offers a more effective acceleration control. This shows the superiority of the HHO-based Type-II Fuzzy control method in terms of increasing passenger comfort via reducing acceleration against the road disturbances. These graphs show that active control methods provide significant performance improvements in vehicle body and acceleration compared to the passive suspension system. In Figure 9 and Figure 10, suspension deflection and tire deflection graphs are presented.

When the suspension deflection (Figure 9a) graph obtained related to the Road 1 input is observed, it is seen that the deflection amplitudes of the HHO-based Type-II Fuzzy control method are lower than the HHO-based Type-I Fuzzy control method. The HHO-based Type-II Fuzzy control method provides a deflection amplitude of approximately 0.021~−0.068 m, while the Type-I Fuzzy control method has a deflection amplitude of approximately 0.037~−0.07 m. When the suspension deflection graph obtained related to the Road 2 input (Figure 9b) is examined, it is observed that the deflection amplitudes of the HHO-based Type-II Fuzzy control method are lower than the HHO-based Type-I Fuzzy control method. While the Type-II Fuzzy control method provides a deflection amplitude in the range of approximately ±0.001 m, the Type-I Fuzzy control method has a deflection amplitude of approximately 0.02~−0.023 m. These results reveal that HHO-based Type-II Fuzzy control controls suspension deflections better. When the tire deflection (Figure 10a) graph obtained related to the Road 1 input is examined, it is observed that the amplitude values of the HHO-based Type-II Fuzzy control type are lower than the HHO-based Type-I Fuzzy control method. While HHO-based Type-II Fuzzy control enables amplitudes ranging from approximately 0.0042~−0.0038 m in terms of tire deflection, the HHO-based Type-I Fuzzy method produces deflection amplitudes between approximately 0.0033~−0.0025 m. This shows that HHO-based Type-II Fuzzy more effectively reduces tire deflection. When the tire deflection graph obtained related to the Road 2 input (Figure 10b) is examined, it is observed that deflection amplitudes of the HHO-based Type-II Fuzzy control method are lower compared to the HHO-based Type-I Fuzzy control method.

**Figure 11:**
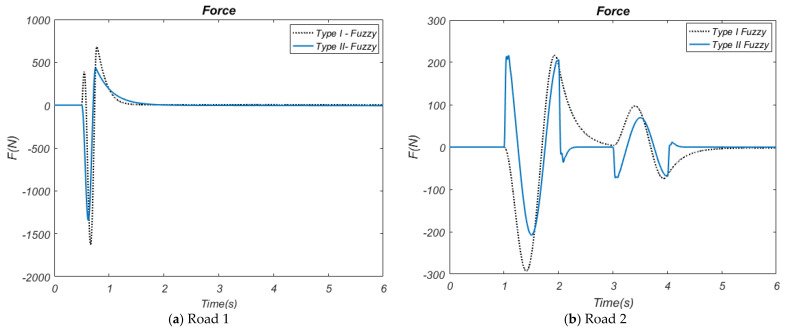
The force.

While the Type-II Fuzzy control method provides a deflection amplitude in the range of approximately 0.0012~−0.0007 m, the Type-I Fuzzy control method has a deflection amplitude in the range of approximately 0.0018~−0.001 m. These results reveal that HHO-based Type-II Fuzzy control controls tire deflections better. In these graphs, it was observed that all active control methods for both road inputs significantly reduced suspension deflection and tire deflection compared to the passive suspension system. In particular, the HHO-based Type-II Fuzzy method increased the overall stability and performance of the system by minimizing deformations on the suspension and tire. The force graphs utilized in active control, illustrated in Figure 11a,b, provide the time-dependent variation in the forces applied by these control methods to the active suspension system.

The controller is more beneficial because it uses less force to reduce vibrations. When the tire deflection (Figure 10a) graph obtained related to the Road 1 input is examined, it is observed that the amplitude values of the HHO-based Type-II Fuzzy control type are lower than the HHO-based Type-I Fuzzy control method. While HHO-based Type-II Fuzzy control provides amplitudes ranging from approximately 480~−1300 N in terms of force, the HHO-based Type-I Fuzzy method produces force amplitudes between approximately 600~−1600 N. When the force graph obtained related to the Road 2 input (Figure 11b) is examined, it is observed that the force amplitudes of the HHO-based Type-II Fuzzy control method are lower than the HHO-based Type-I Fuzzy control method. While the Type-II Fuzzy control method provides a force amplitude in the range of approximately ±205 N, the Type-I Fuzzy control method has a force amplitude of approximately 205~−300 N. These results reveal that HHO-based Type-II Fuzzy control controls tire deflections better. As seen in all graphs obtained in the study, the HHO-based Type-I Fuzzy controller provides more effective suppression of vibrations by using less force. This offers a significant advantage in terms of reducing the vehicle’s energy consumption and increasing the durability of the suspension system. In this study, the comparison of different performance parameters (movement of the vehicle body, vehicle acceleration, suspension deflection, tire deflection, and force to be implemented) of the methods used for both road inputs by using the Root Mean Square Error (RMSE) performance index is given in Table 4 and Table 5. This table provides a numerical evaluation of the effectiveness of the methods and shows that the HHO-based Type-II Fuzzy controller offers the best overall performance.

In Table 4 and Table 5, where the comparison tables are given according to the RMSE performance criteria, the lowest error performance is given in bold characters. In Table 4, the motion and acceleration error results of the vehicle body obtained using the Type-II Fuzzy control method for Road 1 are 0.0086 m and 1.0929 m/s^2^, respectively. 0.0126 m and 1.3821 m/s^2^ belong to the motion and acceleration error results of the vehicle body obtained using the Type-I Fuzzy control method. The suspension deflection and tire deflection error results obtained using the Type-II Fuzzy control method given in the relevant table are 0.0119 m and 0.0009 m, respectively. 0.0163 m and 0.0014 m belong to the suspension deflection and tire deflection results obtained by the Type-I Fuzzy control method. Finally, the force error results presented in Table 4 are 214.4 N and 343 N, respectively. These values belong to Type-II Fuzzy and Type-I Fuzzy control methods, respectively. In Table 5, the motion and acceleration error results of the vehicle body obtained by the Type II Fuzzy control method for Road 2 are 0.0103 m and 0.6226 m/s^2^, respectively. An amount of 0.0172 m and 1.0120 m/s^2^ belongs to the motion and acceleration error results of the vehicle body obtained using the Type-II Fuzzy control method.

**Figure 12:**
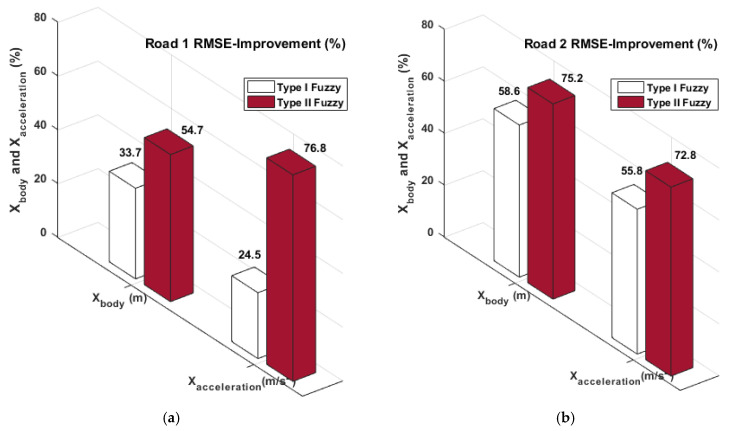
The percentage performance contrasts of the X body position and X body acceleration: (**a**) Road 1 and (**b**) Road 2.

**Figure 13:**
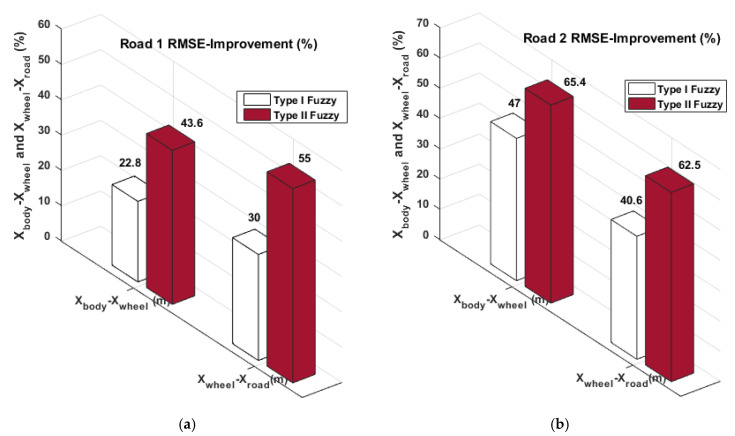
The percentage performance contrasts of the suspension deflection and tire deflection: (**a**) Road 1 and (**b**) Road 2.

The suspension deflection and tire deflection error results acquired utilizing the Type-II Fuzzy control method given in the relevant table are 0.0123 m and 0.0012 m, respectively. An amount of 0.0188 m and 0.0019 m belongs to the suspension deflection and tire deflection results obtained using the Type-I Fuzzy control method. Finally, the force error results presented in Table 5 are 298.76 N and 332 N, respectively. These values belong to Type-II Fuzzy and Type-I Fuzzy control methods, respectively. Equation (26) was used to determine how well the study’s approaches performed compared to the passive system. Figure 12 and Figure 13 show the improvement rates obtained by utilizing the Type-I and Type-II Fuzzy logic control methods.

(26) $$Improvement\left(\%\right)=\left|\frac{passive-active}{passive}\right|\times 100$$

When the improvement graphs presented in Figure 12 are examined, the best improvement rates for Road 1 are the X body position and X body acceleration results obtained by using the Type II Fuzzy control method. These results are 54.7% and 76.8%, respectively. Similarly, the best improvement rates in X body position and X body acceleration for Road 2 are 75.2% and 72.8%, respectively. When the improvement graphs presented in Figure 13 are examined, the best improvement rates for Road 2 are the Suspension deflection and Tire deflection results obtained by using the Type-II Fuzzy control method. These results are 43.6% and 55%, respectively. Similarly, the best improvement rates in X body position and X body acceleration for Road 2 are 65.4% and 62.5%, respectively. Figure 12 and Figure 13 show the improvement rates obtained by using the Type-I Fuzzy control method. When all error results and improvement results obtained by using the RMSE criterion are examined, it is illustrated that the suggested Type-II Fuzzy control method provides more effective suppression of vibrations by using less force and performs better than the Type-I Fuzzy method.

**Figure 14:**
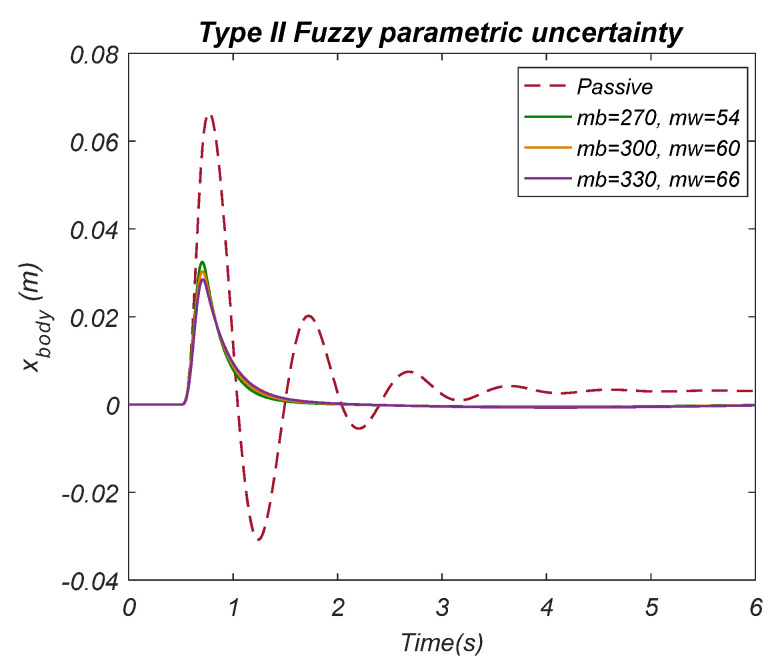
±10 percent parametric uncertainty using the Type II Fuzzy control method.

To indicate the performance of the suggested control (Type II Fuzzy) method, a comparison with the PID control method and the methods used in reference [34] utilizing the integral time squared error (ITAE) criterion to compare with the existing works in the literature is made and given in Table 6 (this comparison was performed using the road input function given in reference [34]). Table 6 compares the error values for tire deflection, suspension deflection, vehicle acceleration, and vehicle body motion with traditional PID, harmonic, and MPC approaches. The corresponding values are underlined to emphasize the lowest error performance values. The suggested Type II Fuzzy control method produces better outcomes than any other method now in use. The performance of the HHO-based Type II control method against ±10 percent parametric uncertainty in the mass of the vehicle body and wheel body is given in Figure 14. In summary, the HHO-based Type II Fuzzy control method was more successful than Type I in reducing vibrations and acceleration fluctuations, thus enhancing vehicle stability and responsiveness. Both methods performed better than passive suspension, suggesting that active control strategies are effective in developing ride comfort. Limitations of the study may include the lack of consideration of environmental factors, road conditions, and driving style; the limited performance of the HHO algorithm in complex systems; and the limited ability to continuously adapt as the model is only valid in certain conditions.

## 5. Conclusions

This paper presents a study in which active control was performed using Type I Fuzzy and Type II Fuzzy control methods with a quarter-vehicle model. The quarter-vehicle suspension system was controlled using two different control methods with two different road inputs. The proposed and implemented controllers were simulated in a computer environment. The results are presented in tables and graphs, and the methods are compared using the RMSE criterion. In addition, the performance of the methods is presented as a percentage compared to the passive suspension system. It was observed that the Type II Fuzzy Logic control method suppresses vibrations more effectively using less force and performs better than the Type I Fuzzy Logic method. All these graphical and numerical results demonstrate significant improvements in road holding and comfort compared to the passive suspension system. However, the current study is limited to a quarter vehicle model and a simulation environment. Real-world complexities such as actuator dynamics, noise, and delays have not been considered. In future studies, the proposed control strategies can be extended to half- and full-vehicle models, and their performance can be validated in experimental and real-time environments. The proposed method can be compared with commonly used optimization methods such as PSO, DE, and ACO, and can also use stochastic road profiles (e.g., ISO class A–D roads) and random road inputs. Furthermore, LQR, SMC, and hybrid control approaches, such as combining Type II Fuzzy Logic with PID control, can be investigated to further enhance system performance and robustness under varying road and load conditions.

## Figures and Tables

**Table 1:** The summary of controllers and optimizers in the literature review.

References	Proposed Control Methods	Optimization Algorithms
Alleyne and Hedrick (1992) [4]	An observer-based nonlinear controller and an adaptive nonlinear controller	
Cherry and Jones (1995) [5]	FLC	
ElMadany and Abduljabbar (1999) [6]	LQG	
D’Amato and Viassolo (2000) [7]	FLC	GA
Foda (2000) [8]	FLC	
Al-Holou et al. (2002) [9]	Sliding mode neural network inference FLC	
Abdelhady and Alhasan (2003) [10]	A neural fuzzy control	
Huang and Lin (2004) [11]	A self-adjusting adaptive control method with a fuzzy control	
Sharkawy (2005) [12]	Fuzzy and Adaptive Fuzzy Control (AFC) methods	
Gao et al. (2006) [13]	PID	
Zhang et al. (2007) [14]	A dynamic sliding-mode controller with fuzzy adaptive tuning	
Cao et al. (2008) [15]	A novel AFC based on interval type-2 fuzzy	
Tusset et al. (2009) [16]	FLC	
Salem and Aly (2010) [17]	FLC	
Lin et al. (2011) [18]	Indirect adaptive interval Type-II fuzzy neural network control sliding mode control	
Bijan et al. (2011) [19]	An interval type-2 fuzzy approach	
Lin and Lia (2012) [20]	A self-organizing fuzzy controller	
Liu et al. (2011) [21]	Reliable fuzzy H∞ control	
Hurel et al. (2012) [22]	FLC	PSO
Kalaivani et al. (2014) [23]	FLC	Hybrid Differential Evolution-based Biogeography-Based Optimization
Emam (2015) [24]	Fuzzy-PID	
Divekar and Mahajan (2016) [25]	Self-tuning fuzzy PID control	
Abougarair and Mahmoud (2017) [26]	Self-tuning fuzzy PID control	
Nagarkar et al. (2018) [27]	PID and Fuzzy control	GA
Lin et al. (2019) [28]	Fuzzy SMC with Proportional Differential Sliding Mode Observer	
Hung et al. (2020) [29]	FLC	
Ahmed (2021) [30]	Fuzzy PID and LQR	
Matrood and Nassar (2021) [31]	Modified PID	
Jibril et al. (2021) [32]	Fuzzy Model Predictive Controller	
Munawwarah and Yakub (2021) [33]	PID-LQR and Fuzzy-PID	
Nichiţelea and Unguritu (2022) [34]	Adaptive harmonic control	
Wang et al. (2022) [35]	Type-2 Fuzzy SMC	
Mahmoodabadi and Javanbakht (2022) [36]	AFC	Gravitational Search Algorithm
Robert et al. (2022) [37]	FLC	
Han et al. (2022) [38]	Adaptive fuzzy PID	
Zhang and Dong (2023) [39]	Nonparallel distribution compensation FLC	
Abut and Salkim (2023) [40]	Fuzzy Linear Quadratic Regulator control	PSO
Nguyen (2023) [41]	Sliding Mode—PID control algorithm tuned by fuzzy method	
Nguyen (2023) [42]	Adaptive Fuzzy–Sliding Mode–Proportional–Integral	-
Zhao and Gu (2023) [43]	LQR	Hybrid particle swarm optimization genetic
Wu and Su (2024) [44]	PID	Q-learning algorithm optimization
Kharola et al. (2024) [45]	Adaptive neuro fuzzy inference system	
Mustafa and Wang (2024) [46]	A new adaptive fuzzy logic control	
Abut et al. (2025) [47]	Fuzzy Linear Quadratic Regulator control	GWO
Lopes et al. (2025) [48]	PID	Giant armadillo optimization algorithm
Zhang et al. (2025) [49]	Neuro adaptive Control	
Yu et al. (2024) [50]	Fuzzy PID	Chaotic PSO
Tang and Ahmad (2025) [51]	FLC	Hybrid water wave and PSO

**Table 2:** The rule table for Tip I Fuzzy, where NB, NS, Z, PS, and PB refer to Negative Big, Negative Small, Zero, Positive Small, and Positive Big, respectively.

$e/\dot{e}$	NB	NS	Z	PS	PB
NB	PB	Z	PS	NS	NB
NS	NB	Z	NS	Z	PS
Z	PS	PS	NS	PS	Z
PS	PS	Z	NB	NB	PB
PB	Z	PS	PS	Z	PB

**Table 3:** The rule table for Tip II Fuzzy.

$e/\dot{e}$	NB	NS	Z	PS	PB
NB	NS	PS	NB	NB	PB
NS	PB	PS	PB	Z	NB
Z	PB	Z	PB	NS	NB
PS	Z	NS	PS	PS	Z
PB	NS	NB	Z	PB	NS

**Table 4:** Comparisons of performance criteria for Road 1.

Performance Criteria (RMSE)	Passive	Type I Fuzzy	Type II Fuzzy
Body travel $\left(X_{body}\right)$ $\left(m\right)$	0.0190	0.0126	**0.0086**
Body acceleration $\left({\ddot{X}}_{body}\right)$ $\left(m/s^{2}\right)$	1.9327	1.3821	**1.0929**
Suspension deflection $\left(X_{body}-X_{wheel}\right)$ $\left(m\right)$	0.0211	0.0163	**0.0119**
Tire deflection $\left(X_{wheel}-X_{road}\right)$ $\left(m\right)$	0.0020	0.0014	**0.0009**
Actuator $\left(F\right)$ $\left(N\right)$	-	343	**214.4**

**Table 5:** Comparisons of performance criteria for Road 2.

**Performance Criteria (RMSE)**	**Passive**	**Type I Fuzzy**	**Type II Fuzzy**
Body travel $\left(X_{body}\right)$ $\left(m\right)$	0.0415	0.0172	**0.0103**
Body acceleration $\left({\ddot{X}}_{body}\right)$ $\left(m/s^{2}\right)$	2.288	1.0120	**0.6226**
Suspension deflection $\left(X_{body}-X_{wheel}\right)$ $\left(m\right)$	0.0355	0.0188	**0.0123**
Tire deflection $\left(X_{wheel}-X_{road}\right)$ $\left(m\right)$	0.0032	0.0019	**0.0012**
Actuator $\left(F\right)$ $\left(N\right)$	-	332	**298.76**

**Table 6:** The Type II fuzzy control approach compared with other approaches.

Performance Criteria (ITAE)	Type II Fuzzy	PID	Harmonic [34]	MPC [34]
Body travel $\left(X_{body}\right)$ $\left(m\right)$	0.0018	0.0052	0.0039	0.0041
Body acceleration $\left({\ddot{X}}_{body}\right)\left(m/s^{2}\right)$	0.0542	0.1830	0.1683	0.1252
Suspension deflection $\left(X_{body}-X_{wheel}\right)$ $\left(m\right)$	0.0019	0.0062	0.0044	0.0046
Tire deflection $\left(X_{wheel}-X_{road}\right)$ $\left(m\right)$	0.0010	0.0082	-	-
Actuator $\left(F\right)$ $\left(N\right)$	51.3	76.2	64	80.7

## Data Availability

All data generated or analyzed during this study are included in this published article.

## Abbreviations

The following abbreviations are used in this manuscript:

**Nomenclature**	
$m_{b}$	Mass of a quarter vehicle body
$m_{w}$	Mass of the wheel assembly
$k_{s}$	Spring coefficient of the suspension system
$b_{s}$	Damper coefficient of the suspension system
$k_{t}$	Spring coefficient of the tire
$b_{t}$	Damper coefficient of the tire
$x_{b}$	Body travel
${\ddot{x}}_{b}$	Body acceleration
$x_{w}$	Displacement of the wheel assembly
$x_{r}$	Road roughness (distortion)
$x_{b}-x_{w}$	Suspension deflection
$x_{w}-x_{r}$	Tire deflection
$F_{s}$	Actuator control force
ASS	Active Suspension System
HHO	Harris Hawks Optimization Algorithm
TypeIFuzzy	Type I Fuzzy Logic
TypeIIFuzzy	Type II Fuzzy Logic
